# Comparison of standard-setting methods for the Korean Radiological Technologist Licensing Examination: Angoff, Ebel, bookmark, and Hofstee

**DOI:** 10.3352/jeehp.2018.15.32

**Published:** 2018-12-26

**Authors:** Janghee Park, Duck-Sun Ahn, Mi Kyoung Yim, Jaehyoung Lee

**Affiliations:** 1 Department of Medical Education, Soonchunhyang University College of Medicine, Asan, Korea; 2 Research institute for healthcare policy, Seoul, Korea; 3 Korea Health Personnel Licensing Examination Institute, Seoul, Korea; Hallym University, Korea

**Keywords:** Licensure, Education, Radiological technologist, Standard setting, Modified-Angoff, Ebel, Bookmark, Hofstee

## Abstract

**Purpose:**

This study aimed to compare the possible standard-setting methods for the Korean Radiological Technologist Licensing Examination, which has a fixed cut score, and to suggest the most appropriate method.

**Methods:**

Six radiological technology professors set standards for 250 items on the Korean Radiological Technologist Licensing Examination administered in December 2016 using the Angoff, Ebel, bookmark, and Hofstee methods.

**Results:**

With a maximum percentile score of 100, the cut score for the examination was 71.27 using the Angoff method, 62.2 using the Ebel method, 64.49 using the bookmark method, and 62 using the Hofstee method. Based on the Hofstee method, an acceptable cut score for the examination would be between 52.83 and 70, but the cut score was 71.27 using the Angoff method.

**Conclusion:**

The above results suggest that the best standard-setting method to determine the cut score would be a panel discussion with the modified Angoff or Ebel method, with verification of the rated results by the Hofstee method. Since no standard-setting method has yet been adopted for the Korean Radiological Technologist Licensing Examination, this study will be able to provide practical guidance for introducing a standard-setting process.

## Introduction

A licensing examination evaluates whether a licensee has appropriate skills in the field after earning a license. The criteria for deciding whether an examinee has the appropriate skills and whether to pass an examinee are very important. The criteria for passing the written component of the Korean Radiological Technologist Licensing Examination (KRTLE) are 60% or higher of the total possible score for all subjects and 40% or higher for each subject.

However, these criteria do not consider the quality and level of difficulty of the items or information about the candidates. In this situation, it can be argued that passing the national examination may not guarantee the minimum competence required by the licensing examination.

In response to this, the Korea Health Personnel Licensing Examination Institute conducted a basic research study to examine possible standard-setting methods [1], and in a recent study, the modified Angoff method was found to be appropriate [2]. National examinations may differ depending on the environment. Currently, the KRTLE is administered once a year, but it could be changed to an examination held several times annually using computerized adaptive testing. Therefore, it is necessary to identify various methods that can be applied to changing examination forms.

The purpose of this study was to apply various standard-setting methods for the KRTLE, which has a fixed cut score, and to suggest the most appropriate standard-setting method.

### Standard-setting methods

Cizek [3] in 1993 defined standard setting as a legitimate and appropriate rule or procedure that assigns numbers to distinguish differences in performance, and emphasized the procedural definition of the standard-setting process.

*Angoff*: Angoff [4] estimated the percentage of correct answers for each item of a minimally competent person belonging to a virtual group through a content analysis of the test tool, tallied up the scores for the total items, and calculated the cut score. The Angoff method is the most widely applied method, and it is easy to explain.

*Ebel*: In the Ebel method, a standard-setting panel first examines each item to determine the level of difficulty (easy, appropriate, and difficult) and relevance (essential, important, acceptable, and questionable) of each item. Then, each item is classified into a 3×4 matrix table according to the level of difficulty and relevance. Next, the panel determines the expected percentage of correct answers to the items in each cell of the matrix table by a person who has minimum competency. Lastly, the number of items in each category is multiplied by the expected percentage of correct answers, and the total results are added to calculate the cut score [5]. The Ebel method involves a more complex standard-setting process than the other standard-setting methods, which are based on an analysis of the content of the test tool, and it therefore imposes a burden on the standard-setting panel [6].

*Bookmark*: The bookmark method was first introduced by Lewis et al. [7] in 1996 as a standard-setting method to calculate the cut score based on the review of a collection of items by standard-setting panelists. This method is called the ‘bookmark’ method because the standard-setting panelists indicate their judgments about a specially created item collection according to the level of difficulty. The specially created item collection is known as the ordered item booklet (OIB). The basic feature of the bookmark method is that it uses item response theory to construct the OIB. The easiest item is placed at the beginning of the OIB, and the hardest item is placed at the end. The advantage of using a scaling method grounded in item response theory is that the item difficulty and the subject’s ability are on the same scale [8,9].

*Hofstee*: Eclectic method of Hofstee [10] in 1983 was developed to address practical problems arising from disagreement between criterion-referenced and norm-referenced predictions. In the Hofstee method, standard-setting panelists answer 4 questions with assumptions about the subjects who first take the test. Two of the questions are about the appropriate level of knowledge that the subjects should have (indicated as k by Hofstee), and the other two are about the fail rate (indicated as f by Hofstee). The questions are as follows: “First, what is the maximum cut score that would be satisfactory, even if all subjects exceed this score? Second, what is the minimum cut score that would be acceptable, even if all subjects do not reach the score? Third, what is the maximum allowable fail rate? Fourth, what is the acceptable minimum fail rate?” [10].

*Selection or allocation of items (subsets)*: A method in which panelists review all the items and determine the cut score takes a great deal of time and effort because of repeated item review and discussion between panelists. Moreover, many items need to be rated, which can reduce reliability. Ferdous and Plake [11] in 2007 introduced and simulated a method to reduce the number of items that the panelists should rate. The first is to evaluate only some of the items by selecting a subset of items for rating. The second is to divide the total items and rate them. When the panelists rated two-thirds of the total items, the results were similar to the results of rating the total items. That the results of rating more than 50% of items were similar to the results of the overall rating. They suggested that items should be selected or allotted based on their content and difficulty [12,13].

## Methods

### Ethical approval

This study was approved by the Institutional Review Board of Korea University (KU-IRB-18-EX-65-A-1). Informed consent was obtained from participants.

### Study design

Descriptive analysis, correlation analysis, and item analysis.

### Materials and/or subjects

The radiological technologist licensing examination was the 44th KRTLE, administered on December 18, 2016. Table 1 shows the number of items and the cut score for each subject. On the written test, candidates must score 40% or more of the total possible score for each subject and 60% or more of the total possible score for all subjects. In the practical skill examination, they must score 60% or more of the total possible score.

The panelists selected in the standard-setting workshop included 6 radiologists who were national examiners. The workshop for setting cut scores for the radiological technologist examinations proceeded from 9 AM to 5 PM on Saturday, May 12, 2018 (Table 2).

The panelists gave feedback from the survey and consultation meeting. At the end of the standard-setting workshop, panelists were surveyed with the following question: “What do you think is the most appropriate standard-setting method for the national examination? Ebel, Angoff, bookmark, or Hofstee.”

An advisory council was held to present the workshop outcomes and the suggested methods for standard setting for the national examinations, and a discussion was held about the following topics: (1) Which is the most reasonable method to apply for the national examination? (2) Do all the panelists have to rate all the items? (3) If items to be rated are divided, what is the proper method for doing so?

### Technical information

In the Angoff method, the panelists reviewed each item and described the expected percentage of correct answers by a minimally competent person. The sum of the expected correct answers for each item was calculated as the cut score.

In the Ebel method in this study, item relevance was classified into the following categories: essential (a task the subject should thoroughly know); important (a major and important task); and additional (an additional task). The expected correct answer rate for a minimally competent person was categorized as hard (50% or less), medium (50%–80%), or easy (80% or higher) [14].

To use the bookmark method, an OIB that arranged items in the order of level of difficulty was prepared in advance. The OIB was produced for each subject area. For the item analysis, the level of difficulty and discrimination were calculated using the R program (https://www.r-project.org/) by applying 2-parameter item response theory. The items were arranged based on the subject’s ability, θ, with a correct answer rate of 0.67 for each item according to the OIB’s production principle. The standard-setting panelists bookmarked the last item that the minimally competent person was expected to answer correctly in each OIB. The competency corresponding to the bookmark point indicated by each panel member was converted into the true score, and the median was determined as the final cut score. The radiologists produced OIBs for 4 subject areas, consisting of 90 items about radiation theory, 20 items about medical regulations, 90 items about radiation application, and 50 items about practical skills.

To apply the Hofstee method, the maximum cut score and minimum cut scores that would indicate competence and the maximum and minimum fail rates were investigated among the panelists, and the average value was used as the final value. Based on the results of the national exam, the cumulative distribution of the fail rate according to the examination score was derived, and the point of intersection with the final score was determined as the cut score.

### Statistics

IBM SPSS ver. 25.0 (IBM Corp., Armonk, NY, USA) was used for the descriptive and correlation analyses, and R ver. 3.4.3 (https://www.r-project.org/) for the item response theory analysis [15]. For the item analysis, the level of difficulty and discrimination were calculated with R by applying 2-parameter item response theory.

## Results

### Definition of a minimally competent person

A minimally competent person was defined as a person who has only worked for 1 day after obtaining the license, and the content of the items and the expected correct answer rate were determined accordingly.

### Comparison of cut scores between cut score setting methods

Table 3 summarizes the results of applying the Angoff, Ebel, bookmark, and Hofstee methods for the KRTLE. Based on a total score of 100, the cut scores assigned by the radiologists were 71.27 using the Angoff method, 62.2 using the Ebel method, 62.49 using the bookmark method, and 62 using the Hofstee method (Appendices 1–4). The cut scores according to the Ebel and bookmark methods were similar, but those according to the Angoff and Hofstee methods were significantly different. For radiologists, the cut score according to the Ebel method was similar to those according to the bookmark and Hofstee methods.

Relationships between standard-setting methods

Table 4 shows the results of confirming the reliability of the rating methods using a correlation analysis by classifying subjects who passed and those who failed according to each cut-off score. The Ebel and Hofstee methods showed similar scores, so the passing and failing rates were similar, too.

### The reliability of the rating method

The reliability of the rating method was confirmed using a correlation analysis by classifying subjects who passed and those who failed according to each cut-off score (Table 5). For the radiologists, the correlation between the Ebel method and the Hofstee method was very high (0.983), as was the correlation between the bookmark method and the Hofstee method (0.917).

Feedback on the standard-setting method

**Fig. 1.**
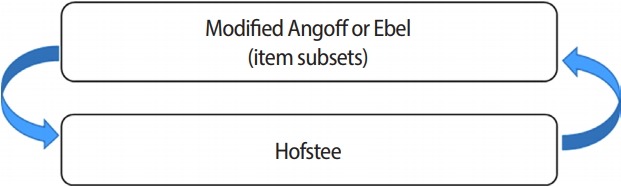
Suggested standard-setting process.

At the end of the standard-setting workshop, a survey was conducted of all 6 panelists. The results for the most appropriate standard-setting method for the national examination were as follows: Ebel, 57.1%; Angoff, 28.6%; Hofstee, 14.3%; and bookmark, 0%. An advisory council was held to present the workshop results and the suggested methods for standard-setting for the KRTLE, along with a discussion about these methods. Four panelists attended, discussed the issues, and decided that they agreed with the suggested standard-setting model proposed in this study and the item subsets (Fig. 1).

Suggestion of a standard-setting method

**Fig. 2.**
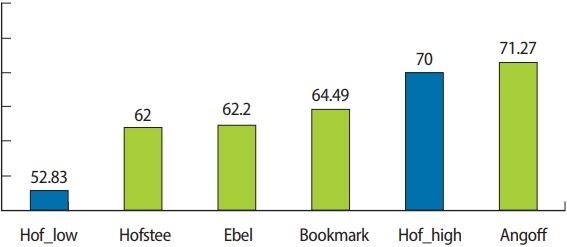
Range of the acceptable low and high cut scores.

The final proposal for a standard-setting method is shown in Fig. 1. In the first step of the standard-setting method, the modified Angoff or Ebel method is used, and in the second step, the Hofstee method is used to check whether the proposed standard-setting method presents an acceptable range of cut scores and fail rates for the national examination. The Hofstee acceptable cut score and fail rate range will not be absolute, but can be used as a reference (Fig. 2).

When using the Angoff method, a modified model that uses test information to set standards will help reduce variation across panelists. For the Ebel method, the test information should be examined, and methods of utilizing the actual level of difficulty should be compared.

Although did not attempt to do so in this study, based on the literature, we suggest that all items should be rated due to the nature of national examinations, and that items should be allocated into subsets according to test subject, test period, and item information. It is appropriate to allocate items according to item information, such as level of difficulty and discrimination.

## Discussion

Standards for the KRTLE were set using the Angoff, Ebel, bookmark, and Hofstee methods. The Ebel and Hofstee methods showed the most similar results, and the cut score according to these 2 methods was also most similar to the current standard of the national examination (a score of 60). Since the cut score of the national examination is fixed, the examination committee members consider the fixed score when developing or organizing national examination items. In other words, the Ebel and Hofstee methods showed the most similar results when assuming that the items were created according to a passing score of 60. The Ebel method comprehensively takes into account the relevance of the items, the expected percentage of correct answers of the minimally competent person, and the percentage of correct answers on items with similar relevance and a similar expected percentage of correct answers by borderline examinees. Thus, the procedure is complicated, but the results were similar to the actual cut-off scores. In this study, the modified Angoff method, which refers to the information of the actual items to set the standard, was not applied. Thus, the cut-off score according to the Angoff method was different from the other cut-off scores.

The standard-setting method proposed in this study is to rate items using the modified Angoff or Ebel methods in the first step and then to confirm the acceptable cut score and fail rate using the Hofstee method. The modified Angoff method, which is the most commonly used method of setting a cut score, and the Ebel method, which yielded relatively stable results in this study, can be applied to obtain the cut score. Then, the Hofstee method is used to examine whether the result is acceptable considering the maximum and minimum ranges of the cut score and fail rate. For the Qualifying Examination part II, which is a practical skill test for doctors in Canada, the cut-off score is calculated using contrasting groups and the borderline group method, and effect of the result is considered through the Hofstee method [16].

While all the panelists evaluated all items in the existing method, we propose the use of item subsets, a partial rating method in which panelists divide the entire set of items and rate them. In this study, partial rating with item subsets was not carried out. However, rating requires considerable time and effort, so if panelists are appropriately trained, the entire item set should be divided and then allocated to panelists. Thus, reviewing and rating only a subset of items would increase the efficiency of the panelists, while maintaining reliability. The panelists who participated in the workshop also mentioned that partial evaluation would be more effective if a sufficient discussion on common items was held. Ferdous and Plake [11] in 2007 set the standard for the ‘No Child Left Behind’ in the United States and asked the panelists to evaluate only some items based on a consideration of their fatigue, which could reduce reliability.

This study is significant, as it applied various standard-setting methods to the KRTLE beyond the existing fixed cut score and proposed a method of combining standard-setting methods for the first time.

## Figures and Tables

**Table 1.** Information on the Korean Radiological Technologist Licensing Examination

Subject	No. of items	Allocation	Period
Radiation theory	90	90	1
Medical regulations	20	20	
Radiation application	90	90	2
Practical skills	50	50	3
Total	250	250	

**Table 2.** Schedule of the standard-setting workshop

Time	Content
9:00–09:30	Introduction of the standard-setting methods
9:30–10:30	Angoff & Ebel: rating of individual item of total items, agreement by group
10:30–12:00	Angoff & Ebel: total items, agreement of all panelists
12:00–13:00	Lunch
13:00–15:00	Angoff & Ebel: total items, agreement of all panelists
15:00–15:40	Bookmark
15:40–16:10	Hofstee
16:10–16:30	Survey of panelists
16:30–17:00	Discussion

**Table 3.** Comparison of the derived cut scores according to the standard-setting method

	Cut-off score	Total score of 100
Angoff	178.17	71.27
Ebel	155.5	62.2
Bookmark	161.23	64.49
Hofstee	155	62
Total	250	100

**Table 4.** Classification of subjects who passed and those who failed according to the cut-off score

Pass or fail	Angoff	Ebel	Bookmark	Hofstee
Fail	970 (37.0)	532 (20.3)	593 (22.6)	532 (20.3)
Pass	1,652 (63.0)	2,090 (79.7)	2,029 (77.4)	2,090 (79.7)

Values are presented as number (%).

**Table 5.** Correlations between the cut score setting method and whether subjects passed

	Angoff	Ebel	Bookmark
Ebel	0.658		
Bookmark	0.706	0.932	
Hofstee	0.647	0.983	0.917

## Supplementary materials

Supplement 1. Audio recording of the abstract.

## Appendix 1. Result of Ebel method

**Table:**

Relevance	Level of difficulty	No. of questions (A)	Expected rate of correct answers (B)	A×B
Essential	Easy	4	0.8	3.2
	Medium	125	0.7	87.5
	Hard	1	0.5	0.5
	Subtotal	130		91.2
Important	Easy	0	0.7	0
	Medium	44	0.6	26.4
	Hard	1	0.4	0.4
	Subtotal	45		26.8
Additional	Easy	2	0.6	1.2
	Medium	72	0.5	36
	Hard	1	0.3	0.3
	Subtotal	75		37.5
Total				155.5/250
				62.2/100

## Appendix 2. Result of bookmark method

**Table:**

Subject panel	Radiation theory			Medical regulation			Radiation application			Practical skills			Total score	Pass (%)
	OIB	Ability	Score	OIB	Ability	Score	OIB	Ability	Score	OIB	Ability	Score		
P1	47	-0.92	55	11	-1.46	13	44	-0.99	59	30	-0.52	35	161.35	76.60
P2	46	-1.10	51	14	-1.05	15	42	-1.02	58	30	-0.52	35	159.33	77.70
P3	50	-0.86	56	12	-1.21	14	42	-1.02	58	25	-0.70	33	160.87	76.60
P4	50	-0.86	56	13	-1.07	15	47	-0.82	63	27	-0.58	35	167.58	71.70
P5	47	-0.92	55	10	-1.50	12	45	-0.95	60	26	-0.65	34	160.54	76.60
P6	46	-1.10	51	12	-1.21	14	45	-0.95	60	22	-0.74	33	157.68	78.30
Cut-off score													160.71	76.60

OIB, ordered item booklet.

## Appendix 3. Result of Hofstee method

**Table:**

Relevance	Range	Mean ± standard deviation	Final
Fail rate (%)	Maximum	30.00 ± 3.16	20.00
	Minimum	11.17 ± 2.04	
Cut score	Maximum	70.00 ± 7.07	62.00
	Minimum	52.83 ± 6.01	

## References

[1] Park HK (2006). Study on setting a passing score on Korean National Medical Licensing Examination.

[2] Yim M (2018). Comparison of results between modified-Angoff and bookmark methods for estimating cut score of the Korean medical licensing examination. Korean J Med Educ.

[3] Cizek GJ, Bunch MB (2007). Standard setting: a guide to establishing and evaluating performance standards on tests.

[4] Angoff WH (1984). Scales, norms, and equivalent scores.

[5] Thorndike RL, Angoff WH (1971). Educational measurement.

[6] Ebel RL (1972). Essential of educational measurement.

[7] Mitzel HC, Lewis DM, Patz RL, Green DR, Cizek GJ (2001). The bookmark procedure: psychological perspectives. Setting performance standards: concepts, methods, and perspectives.

[8] Skaggs G, Tessema A (2001). Item disordinality with the bookmark standard setting procedure.

[9] Kim NJ (2010). The standard setting of clinical performance examination (CPX) by modified Angoff, bookmark, and item-descriptor matching (IDM) method.

[10] Hofstee WK, Anderson SB, Helmick JS (1983). The case for compromise in educational selection and grading. On educational testing.

[11] Ferdous AA, Plake BS (2007). Item selection strategy for reducing the number of items rated in an Angoff standard setting study. Educ Psychol Meas.

[12] Ferdous AA, Plake BS (2005). The use of subsets of test questions in an Angoff standard-setting method. Educ Psychol Meas.

[13] Sireci SG, Patelis T, Rizavi S, Dillingham AM, Rodriguez G (2000). Setting standards on a computerized-adaptive placement examination.

[14] Lee YJ, Park JH, Cheong JW, Kim SJ, Kim YD (2017). Study on standard setting methods applied to medical school class. J Educ Cult.

[15] Partchev I (2012). Simple interface to the estimation and plotting of IRT models: R package version 0.1.6 [Internet]. http://cran.r-project.org/web/packages/irtoys.

[16] Psychometrics and Assessment Services (2015). Technical report on the standard setting exercise for the medical council of Canada qualifying examination part II [Internet]. https://mcc.ca/media/MCCQE-Part-II_Standard-Setting-Report_July-2015.pdf.

