# Supplementary figures and images for: Comparison of standard-setting methods for the Korean Radiological Technologist Licensing Examination: Angoff, Ebel, bookmark, and Hofstee
Source: J Educ Eval Health Prof. 2018 Dec 26;15:32. doi: 10.3352/jeehp.2018.15.32 (PMC6380908; doi:10.3352/jeehp.2018.15.32)

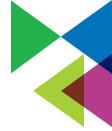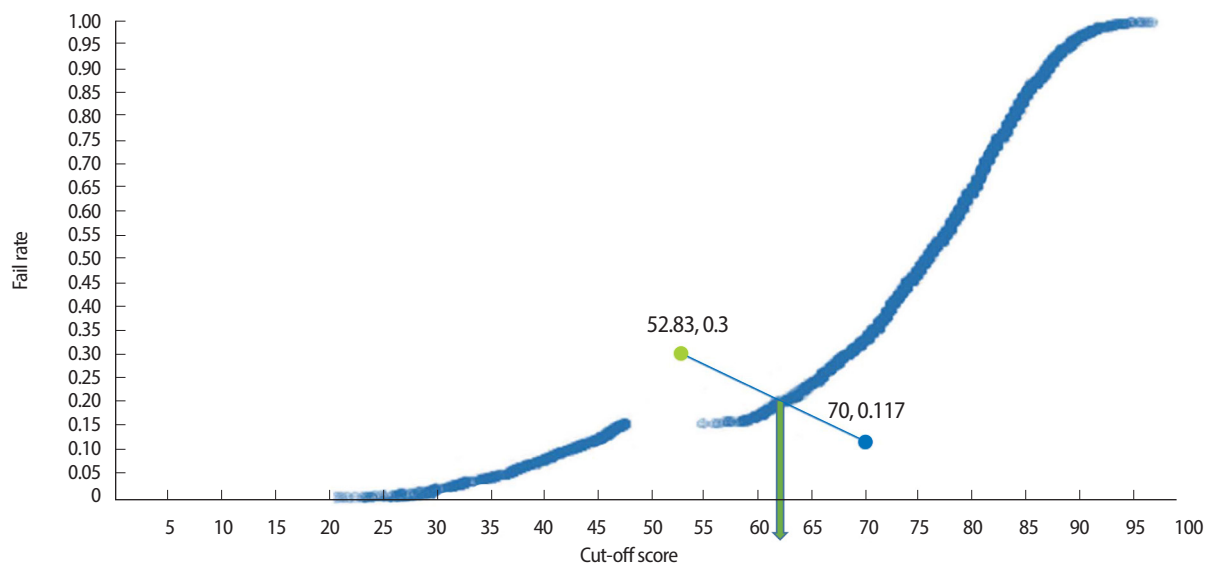

Appendix 4. Result of Hofstee graph.

Supplement: Supplementary file 2 [file jeehp-15-32-app.pdf]
